# Lightning Rods

**Published:** 1845-10

**Authors:** 


### LIGHTNING RODS.

Hitherto, in the Western States, the lightning rod has been very generally neglected, whether because accidents by lightning have not been so frequent as to arrest public attention, or because the efficacy of the conductor is distrusted by the public, we do not at present propose to inquire; but the fact is, that in town and country comparatively few houses will be found thus secured. We incline to believe that the value of the protector is gaining upon the confidence of the people, and to answer the frequent inquiries made of us as to the best mode of erecting the rod, we have determined to write a short article on the subject.

In a letter which we received some time since, the following questions among others were asked:—

"Is there any advantage in having the rod painted black, or any other color? Should the rod be fixed firmly to the top of the chimney, or would a slight motion affect its conducting power? Will the fluid take a lateral direction from the rod if properly constructed; and is there any additional security in having the point made of solid silver? The house being 28 feet long, with a chimney at each end, how far ought the rod to extend above the highest part of the building, and would there be an advantage in having the rod in the centre, or in having two rods? Would the protection be greater from having the two rods connected by a cross wire or rod along the comb of the house, or would such an arrangement be safe? Is there any such thing as the lightning being drawn down between two rods, or any superior efficacy in a number of rods affixed to the several chimneys of an extensive building? Some recommend copper rods, but would not this metal be melted by a heavy charge?"

The writer goes on to say that he has "a rod by the side of his chimney rising 10 feet above it, plated at the point, going 12 feet in the ground to permanent moisture, not painted, and fastened to the chimney by wooden cleats." He asks whether "it would be safer to take it from the chimney, and erect it at the side of the bulding, or to put another at the other end, and if so whether it would be best to have them connected by a rod along the roof?"

A short extract from Daniell's Introduction to Chemical Philosophy answers most of these questions, and as it gives what doubtless is a true account of the *modus operandi* of the lightning rod, we suppose we could not do better than to give it in this place. He says—

"An objection, founded on ignorance, has sometimes been made to lightning rods, that they may, by their attractive power, invite a discharge where otherwise it would not have taken place. But when it is considered that one of the conditions of a thunder storm is an intense electrical induction of a portion of the earth's surface through a dry stratum of air to the surface of a cloud of some thousands of acres in extent, in the manner of a Leyden jar, it will be perceived that any such elevation as compared with the distance and extent of the charged clouds, is utterly inconsiderable, and perfectly inadequate to influence the charge in the slightest degree. The conductor is perfectly passive, and its efficacy consists in opening an easy path by which the force may be transmitted. Its action is but at best of a negative kind, and it can no more be said to attract the lightning than a water-course can be said to attract the water which necessarily flows through it at the time of heavy rains."

In Franklin's works these objections are stated at length as they were formally laid before the Royal Society of London by one of the philosopher's cotemporaries. They will be read with interest as a part of the history of electricity.

"Mr. Benjamin Wilson, one of the committee appointed by the Royal Society of London, to inquire into the propriety of fixing conductors for securing the powder magazines at Purfleet, from lightning, dissented from the opinion of Franklin, Cavendish, Watson, and Robertson, the other members, as to the effect of *pointed* conductors. His words are, 'I dissent from the report in that part only which recommends that each conductor should terminate in a *point.*

"'My reason for dissenting is, that such conductors are, in my opinion, less safe than those which are not *pointed.*

"'Every *point*, as such, I consider as *soliciting* the lightning, and, by that means not only contributing to *increase* the quantity of every actual discharge, but also frequently occasioning a discharge, where it might not otherwise have happened.

"'If, therefore, we invite the lightning while we are ignorant what the quantity or the effects of it may be, we may be *promoting* the very mischief we mean to prevent.

"'Whereas, if instead of pointed, we make use of blunted conductors, those will as effectually answer the purpose of conveying away the lightning *safely*, without the tendency to *increase* or *invite* it.'"—*Franklin's Works, vol. V., p.* 434. *See Philos. Trans. vol, LIV. p.* 247.

The view of Prof. Daniell very much simplifies the whole affair of the lightning rod. It is, that it acts by "*opening an easy pathway*" for the electrical fluid. What is requisite then is, that the rod should be large enough, without any break from top to bottom, and terminate in a good conductor below. If there be any interruption in it, the fluid will pass with less facility, leaping from one broken end to the other, instead of flowing on in a continuous current. And so, too, if the termination below be in a dry bed of clay or sand, the charge cannot escape readily, and the power of the rod is greatly impaired. Water is the best conductor that offers in most situations, and its power is computed to be two hundred thousand times less than that of iron. In cities where there are gas or water pipes, the best possible conditions exist for rendering the rod effectual, for connected with a mass of metal extending miles under ground, it would offer the most perfect door to the fluid that could be imagined.

We are not sure that the importance of this point has not been exaggerated; certain it is, at least, that houses have been protected by rods erected in the clumsiest possible manner. In one case the charge passed down a rod which had been broken several feet from the ground, and from the extremity suspended in the air leaped to the earth. In another case the tin gutter and pipe conveyed the fluid to the ground, taking a man who chanced to be near in its progress. This example is instructive. At the sound of thunder this individual went to the water barrel to place it under the spout, and was standing near the spout when the chimney of the house was struck. The fluid entered his body through his watch, which was in his vest pocket, and descended into the earth. He was severely stunned, but recovered from all alarming symptoms in a few minutes. The chimney was a good deal shattered, and some of the shingles torn up, but the whole charge passed down the tin gutters, and the persons in the house escaped unhurt.

Assuredly it is safer to have a good conductor at the end of the rod in the ground, but these facts, we think, are sufficient to show, that a rod which has no better termination than the common earth is not wholly inefficacious. We have been inclined to attach but little consequence to the termination in points above; but from a communication recently in the newspapers, we see that Professor Hare is disposed to insist upon them. "Under ordinary circumstances," he says, "the competency of lightning rods is dependent on the pointed form given to the upper end, which prevents the electricity from being received above in greater quantity than it can get off through the soil below."

Upon the view given by Daniell, which we adopt, we do not perceive how the point can exercise much influence; at the same time we would not discourage the common practice of furnishing good points to the conductor, which may be attended by greater security. Professor Hare, in the same communication, urges the importance of attaching the lightning rod to the "westernmost part, and preferably the northwesternmost end or corner of the building." He adds, "I am under the impression that all thunder-gusts, in this part of the world, come from the westward. We have sometimes storms from the southwest, accompanied by diffuse electrical flashes, but such genuine thunder-gusts as produce dangerous discharges of concentrated lightning, agreeably to my observations, come always as above stated."

Coils of copper wire, half an inch in thickness, are used in Germany, and as copper is a better conductor than any of the less costly metals it affords, perhaps, the most desirable material, but iron is good enough. And as to number, it may be said, the more the better. It has been recommended to connect the lightning rod with the tin gutters, and the suggestion is unquestionably a good one. A house covered with a metallic roof, and having a sufficient number of conductors going into water, or connected with gas or water pipes, we have been in the habit of saying, would be *lightning proof.* The usual directions given are to extend the rods about ten feet above the chimney. For a small building one rod is probably sufficient, but for powder magazines, and for buildings of great extent, two or more would certainly increase the safety.

